# A Stage 1 Pilot Cohort Exploring the Use of EMDR Therapy as a Videoconference Psychotherapy During COVID-19 With Frontline Mental Health Workers: A Proof of Concept Study Utilising a Virtual Blind 2 Therapist Protocol

**DOI:** 10.3389/fpsyg.2022.901855

**Published:** 2022-07-06

**Authors:** Derek Farrell, Anastasia Fadeeva, Zeynep Zat, Lorraine Knibbs, Paul Miller, Ian Barron, Helga Matthess, Cordula Matthess, Neta Gazit, Matthew D. Kiernan

**Affiliations:** ^1^Department for Violence Prevention, Trauma and Criminology (VPTC), School of Psychology, University of Worcester, Worcester, United Kingdom; ^2^Northern Hub for Veteran and Military Families’ Research, Northumbria University, Newcastle upon Tyne, United Kingdom; ^3^Mirabilis Health Institute, Newtownabbey, United Kingdom; ^4^Centre for International Education, College of Education, University of Massachusetts, Amherst, MA, United States; ^5^remotEMDR, Arbel, Israel

**Keywords:** EMDR therapy, pathogenic memory, adverse and benevolent childhood experiences, videoconference psychotherapy, Blind 2 Therapist

## Abstract

**Objective:**

The COVID-19 pandemic has had a major impact on the delivery of psychological treatment. Due to social distancing requirements, the provision moved to videoconferencing psychotherapy (VCP). There is a paucity of empirical data supporting the efficacy of EMDR therapy as a VCP. This stage 1 pilot study tested an EMDR therapy scripted protocol, such as Virtual Blind 2 Therapist (VB2Tr), on frontline mental health workers as a VCP regarding fitness for purpose, distinctiveness, relevance, and efficiency.

**Methods:**

A total of 24 participants were recruited for the study. The design included a one-session treatment intervention with pre, post, 1-month, and 6-month follow-up (FU) measurements. This treatment session used a “Blind 2 Therapist” EMDR therapy scripted protocol as videoconference psychotherapy that involves non-disclosure of traumatic memory. The research explored the treatment effect on the core characteristics of trauma memory, including subjective disturbance, belief systems, memory intensity (MI), vividness, and levels of emotionality. Additionally, the research explored participants’ experiences of adverse and benevolent childhood experiences (ACEs/BCEs) during their childhood.

**Results:**

Regarding the four tests, namely, fitness for purpose, distinctiveness, relevance, and efficiency, results are favourably suggesting potential clinical benefits of using EMDR as videoconference psychotherapy. Although this is a proof-of-concept study showing positive results, no clinical population or control group was used. The purpose of the study is to explore the potential for scalability toward a larger clinical trial. The treatment intervention was achieved irrespective of either ACEs/BCEs during childhood.

**Conclusion:**

The research tentatively supports the case for EMDR therapy as a credible treatment when used as video conference psychotherapy and in using the Blind 2 Therapist protocol. However, more research is needed to scale toward a clinical trial.

**Clinical Trial Registration:**

**Clinical Trial Registration:** https://www.isrctn.com/ISRCTN12099530, identifier ISRCTN12099530.

## Introduction

In an attempt to reduce the risk of infections from COVID-19, many mental healthcare providers are closing their doors to patients requiring face-to-face therapy and instead of creating videoconferencing psychotherapy (VCP), remote access technology, e-health tools, and Internet interventions (Turgoose et al., 2018; Wind et al., 2020a). Several comprehensive reviews highlight the effectiveness of videoconferencing psychotherapy (VCP) and therapist-guided interventions for conditions such as anxiety, major depressive disorders, and trauma (Anderson et al., 2003; Ruskin et al., 2004; Christopher Frueh et al., 2007; Germain et al., 2010; Osenbach et al., 2013; Rousmaniere et al., 2014; Berryhill et al., 2018, 2019; Karyotaki et al., 2018a,b; Tuerk et al., 2018; Turgoose et al., 2018; Watts et al., 2020; Weinberg, 2020a; Wind et al., 2020b). Contemporary research supports VCP as “feasible” and “acceptable” as a mode of psychological treatment delivery, providing high satisfaction and effectiveness (Simpson and Reid, 2014; Dores et al., 2020; Viswanathan et al., 2020; Weinberg, 2020b; Wells et al., 2020; Wind et al., 2020b; Wright and Caudill, 2020; Aafjes-van Doorn et al., 2021; Hoffman, 2021). Furthermore, VCP provides a viable alternative to providing continuity of care in times of social, economic, and health upheaval (Crowe et al., 2020).

There are several distinct barriers toward the equitable provision and access to evidence-based, face-to-face/in-person psychological treatments, with a paucity of suitably qualified mental health workers to sufficiently address the global burden of mental illness and psychological trauma (Farrell et al., 2020). With relatively few providers trained in the therapies underpinned by a solid empirical evidence base, those who live in rural or remote communities are further restricted. The barriers to care are compounded further by disability, poverty, and stigma (Myers et al., 2008; Morland et al., 2011; Tuerk et al., 2018). VCP provides alternative flexibility and equity of access than in-person therapy, with potential for financial efficiencies and cost savings, enhanced reach, flexible implementation, improved cultural adaptability and sensitivity, and improved equity when compared with in-person therapy (Barak et al., 2008; Morland et al., 2011; Backhaus et al., 2012; Bolton et al., 2014; Wild et al., 2016; Baños et al., 2017).

Despite potential benefits, VCP does require critical consideration. One factor is the impact that VCP has on the therapeutic alliance when assuming the “In person,” traditional model, which is the gold standard for psychotherapy. Recent studies (Germain et al., 2010; Simpson and Reid, 2014; Berger, 2017) acknowledge that an effective therapeutic alliance is essential in underlying successful therapy. Table 1 outlines the advantages and disadvantages of using V and how they can be adapted to promote greater effectiveness (Dores et al., 2020; Viswanathan et al., 2020; Weinberg, 2020b; Wells et al., 2020; Wind et al., 2020b; Wright and Caudill, 2020; Aafjes-van Doorn et al., 2021; Hoffman, 2021).

**TABLE 1:** Advantages, disadvantages, and adaption factors in VCPs.

Advantages	Disadvantages	Adaptation factors to promote greater effectiveness
° Greater flexibility ° Cultural adaptability ° Enhanced reach ° Better use of scarce resources ° Cost efficiencies ° Increased accessibility ° No geographical restrictions ° Environmentally – reduces carbon footprint ° Responds to the need for rural services for veterans ° Convenience and affordability for disabled people	° Technology knowledge, application, functioning, and reliability, including challenges ° Poor internet connections, particularly in low socioeconomic areas ° Body language restricted to head and face ° Privacy into the home environment ° Creating safe space, time, and relationships ° Cultural considerations and norms ° Risk management & triage ° Geographical factors, legislation, professional indemnity, logistics ° Insurance cover and liability	° Adjusting for more restricted access to non-verbal communication ° More regular “checking in” with clients ° Requesting more information and clarification on specific points ° More focus on facial expressions and bodily gestures ° More frequent emotion checking ° Enhanced preparation before sessions ° More control of the space ° The therapists assuming a greater sense of ownership and responsibility for the therapeutic alliance within the therapy work ° Client centredness – the Therapist “tailoring” their approach more to the client individual and specific needs

The reality of the COVID-19 pandemic ostensibly has removed choice for a great many individuals, with the options being VCP intervention, no intervention, or an extensive and uncertain period of waiting. The current advances in VCP technology enable it to offer an innovative solution as a viable alternative to in-person therapies.

The World Health Organisation (2020) has expressed concern over the psychological impact of the pandemic and social distancing on the mental health of a broad sector of society. The psychosocial consequences include increases in loneliness, anxiety, depression, gender-based violence, insomnia, substance misuse, self-injurious activity, and suicidal behaviour (Cullen et al., 2020; Ghebreyesus, 2020; Kavoor, 2020; Khan et al., 2020; Kumar and Nayar, 2020; Talevi et al., 2020; Usher et al., 2020). However, COVID-19 is not “ground zero” when considering the mental well-being of a population. Those clients with pre-existing mental health issues before the pandemic started risk further minimisation of their lived experience, and potential to fall out’ of existing service provision. It is essential to acknowledge that events that pre-date COVID-19 may still influence an individual’s response. Two considerations are exposure to adverse childhood experiences (ACEs) and benevolent childhood experiences (BCEs). Exposure to ACEs is the single most potent global public health issue when considering social inequality, lifelong impact on health and behaviour, and social deprivation. Arguably the COVID-19 pandemic further compounds antecedent ACEs. Social inequalities such as these create significant barriers when accessing services, either in person or via VCP (Felitti et al., 1998; Brown et al., 2009; Kim et al., 2010; Burke et al., 2011; Bellis et al., 2014a,b, 2015, 2017, 2019; Islam et al., 2021).

Videoconferencing psychotherapy interventions have primarily focussed on prolonged exposure (Strachan et al., 2012; Hernandez-Tejada et al., 2014; Yuen, 2015; Acierno et al., 2016; Clapp et al., 2016; Acierno, 2017; Tuerk et al., 2018), behavioural activation (Luxton et al., 2015; Acierno et al., 2016; Acierno, 2017), cognitive processing therapy (Morland et al., 2011; Fortney et al., 2015; Grubbs et al., 2015; Maieritsch et al., 2016), and CBT (Olthuis et al., 2016a,b). Currently, limited research publications support EMDR therapy, which uses VCP to treat post-traumatic stress disorder (PTSD) (Todder et al., 2007; Todder and Kapln, 2007; Lightstone et al., 2015). However, a recent study (Bongaerts et al., 2021) has used home-based psychotherapy, delivered by telehealth, as a treatment intervention for complex PTSD. The intervention was delivered in an intensive format, offering both prolonged exposure and eye movement desensitisation and reprocessing [EMDR therapy]. Six participants took part in the study, with two-thirds losing their PTSD or complex-PTSD diagnostic status, demonstrating that the telehealth intervention was both safe and effective (Bongaerts et al., 2021). However, the sample size in their study was small, with just six participants, and only four of the six losing their diagnosis. The safety and effectiveness determinants of the EMDR therapy intervention indicate more extensive and more representative sample sizes.

Eye movement desensitisation and reprocessing therapy, an empirically supported intervention for PTSD and complex PTSD (Christopher Frueh et al., 2007; American Psychological Society, 2017; ISTSS, 2018; Bisson et al., 2019; de Jongh et al., 2019b; Karatzias et al., 2019, 2020), was developed in the late 1980s by an American psychologist, Francine Shapiro. Its primary foci are on the treatment of pathogenic memories and their associated stress symptoms using the model of pathogenesis and change known as adaptative information processing (Hase et al., 2017; Valiente-Gómez et al., 2017). Shapiro (2018) considered trauma memories in a range of mental health disorders and not just PTSD and complex PTSD. Within the AIP theoretical framework, a meta-theory unique to EMDR therapy, the model assumes that the human brain can usually process memories of adverse life events to complete integration. The essence of EMDR therapy involves four distinct aspects, namely, preparation, access, stimulation, and integration [PACI]. What gives EMDR therapy a specific distinctness relates to bifocal physical stimulation, a working memory taxation device that enables the client to attend to internal and external stimuli (de Jongh et al., 2019a,b, 2020). The hypothesised working mechanism of EMDR therapy is still under investigation (Matthijssen et al., 2021). However, most evidence supports the working memory account. Working memory has a limited capacity. Therefore, dual taxation sets up a competing situation. Consequently, the emotional intensity of the pathogenic memory, with all its subjective levels of disturbance, is gradually lost and eventually reconsolidated into a less disturbing and reduced emotional form. Within the EMDR therapy literature, the dominant empirical evidence supports physical eye movements; however, other forms of bifocal physical stimulation can include acoustic, somatic, or multiple forms such as is used within EMDR 2.0 (de Jongh et al., 2020).

The core characteristics of the EMDR B2T protocol are to access and activate a pathogenic memory. However, the primary distinction between this protocol and the standard protocol is that the client does not reveal details about the memory itself, other than its emotional and somatic content and an indication of their subjective unit of distress (SUD). Clients are not under pressure to disclose any of the trauma content during trauma processing using B2T. Table 2 highlights the core components of the EMDR therapy B2T, its context regarding the eight phases of therapy, and the assessment phase (Phase 3 of the standard protocol of EMDR therapy).

**TABLE 2:** EMDR Blind 2 Therapist protocol in context, adapted from Wolpe and Lazarus (1966), Shapiro (2018, 1995, 2001), and Farrell et al. (2020).

EMDR therapy: 8 phases	EMDR therapy Phase 3 Standard Protocol – Assessment Structure	EMDR therapy Phase 3 Assessment Blind 2 Therapist structure and VB2Tr version
Phase 1: history taking	Target Memory	Target memory – cue word
Phase 2: preparation	Worst Image	Emotions
**Phase 3: assessment**	Negative Cognition	Subjective Unit of disturbance (SUD 0–10)
Phase 4: desensitisation	Positive Cognition	Location of body sensation
Phase 5: installation	Validity of Cognition (1–7)	
Phase 6: body scan	Emotion	
Phase 7: closure	Subjective Unit of Disturbance (0–10)	
Phase 8: re-evaluation	Location of body sensation	

Many empirically supported treatments for PTSD contain various elements and degrees of exposure. These rely upon the client’s ability and willingness to disclose the memory of the adverse life event causing a stress response. When pathogenic memories involve shame, guilt, disgust, fear of retribution, lack of language, and non-disclosure self-protection/preservation factors, disclosure may not even be a viable option for a client. A study published from research carried out in Northern Iraq tested the “Blind 2 Therapist” (B2T) protocol, an adaption of EMDR therapy. This study demonstrated the safety, effectiveness, efficiency, and relevance as a treatment intervention for both “shame-based” and “fear of retribution” trauma (Farrell et al., 2020). However, an evaluation of EMDR B2T as a VCP method of delivery has not occurred to date. Therefore, this study aimed to test the virtual version of B2T (referred to as VB2Tr), delivered as a VCP, as a suitable clinical intervention in the desensitisation and reprocessing of a pathogenic memory.

As indicated earlier, within the existing EMDR therapy literature, there is a paucity of research regarding the use of VCP EMDR (Bongaerts et al., 2021). In addressing this aspect, any potential study would have to address two significant aims:

1. What adaptations, if any, would be required to use EMDR therapy as a VCP?
2. Do we critically consider the potential advantages/disadvantages of EMDR therapy as a VCP?

Therefore, the purpose of this study was to determine whether the application of VCP EMDR therapy would be associated with (1) fitness for purpose – safe and effective, (2) distinctiveness – alterations in the core components of a pathogenic memory regarding intensity, vividness, and emotionality, (3) relevance, and (4) efficiency.

A directional hypothesis predicts a positive or negative change between two variables in a specific population. These changes were measured at pre, post, 1-month, and 6-month FUs. The research study hypotheses were as follows:

Hypothesis 1: Fitness for purpose – Virtual Blind 2 Therapist EMDR (VB2Tr), when delivered as VCP, will have no impact on the SUD and the validity of cognition (VOC) regarding a pathogenic memory when measured at post-treatment, 1-month, and 6-month in comparison to a pre-measure.

Hypothesis 2: Distinctiveness – VB2Tr, as a VCP, will have no impact on reducing MI, memory emotionality (ME), and memory vividness (MV) of a pathogenic memory following intervention when measured at post-treatment, 1-month, and 6-month in comparison to a pre-measure.

Hypothesis 3: Relevance – when using VB2Tr, ACEs or BCEs will influence the processing of a pathogenic memory.

Hypothesis 4: Efficiency – as VB2Tr takes longer in time than the 60–90 min recommended by www.emdria.org, it would be more expensive as a clinical intervention.

## Methodology: Research Design

Ethical approval for the study was granted by the University of Worcester (United Kingdom) [CBPS1920031-R2]. Consequently, all the methods used for the study were carried out in strict adherence to the ethical approval granted and in accordance with relevant guidelines and regulations. Informed consent was obtained from all subjects. Additionally, the study was registered as a clinical trial ID ICSRCTN12099530 [30/06/2021]. This stage 1 pilot study used a pre-test/post-test design for taking measures before and after a one-session treatment using the EMDR VB2Tr protocol, including 1-month and 6-month FUs to determine the impact of the treatment intervention on the pilot cohort. The rationale for an experimental design as a stage 1 research project was to determine proof of concept before proceeding to stage 2, involving a quasi-experimental design utilising a distinct control group. The longer-term strategy is for phases 1 and 2 to support a more significant funding application, utilising a randomised control design incorporating a delayed treatment paradigm.

### Participants

As this was a “proof of concept” study, and consistent with the COVID-19 focus, the research study participants were frontline, mental health workers engaged in active clinical practice during the first period of lock down in the United Kingdom. The design of the study incorporated a self-selecting (volunteer) sampling approach, with participants recruited via advertising on psychotherapy Internet forums. Inclusion criteria were as follows:

• Clinically active using online treatment platforms – remote working.
• Encountered an adverse life event that generated a presently held, subjective level of disturbance (SUD+).
• Willingness to be a client for a one-session intervention using the EMDR therapy Blind 2 Therapists protocol (VB2Tr) as a VCP, using the remotEMDR platform.
• No expectation to disclose anything about the target adverse memory.

A sample size of 17 was deemed sufficient to compare findings from the original study, which used the EMDR Blind 2 Therapist protocol with participants from Northern Iraq (Bennett-Levy and Lee, 2014; Chigwedere et al., 2020; Collard and Clarke, 2020; Farrell et al., 2020; Scott et al., 2021). A total of *N* = 24 was recruited for the study.

### Measures Used for the Study

The pre-test/post-test design utilised the following measures:

• Subjective unit of disturbance (SUD) is a 0–10 scale for measuring subjective levels of distress or disturbance currently experienced by an individual (Wolpe and Lazarus, 1966).
• The validity of the cognition scale (VOC) provides a rapid assessment of cognitive structure on an emotional/somatic level rather than an intellectual level (Shapiro, 2018, 1995, 2001). Both the SUD and VOC have documented validity, reliability, and correlations with several physiological indices of distress.
• Memory vividness (MV) and emotionality (ME) – a subjective unit of measurement (0–10) of the vividness of the target memory, either positive or negative (Andrade et al., 1997; Gunter and Bodner, 2008; Maxfield et al., 2008; Engelhard et al., 2010; Schubert et al., 2011, 2016; van den Hout et al., 2011; Leer and Engelhard, 2020).
• Memory intensity (MI) is a subjective unit of measurement (0–10) of the intensity of the target memory, either positive or negative (van der Kolk, 2003, 2007, 2015).
• Adverse childhood experience scale (ACE) collects crucial information based upon the prevalence of adversity during childhood in ten categories before the age of 18: emotional abuse (recurrent), physical abuse (recurrent), sexual abuse (contact), physical neglect, emotional neglect, substance misuse in the household, mental illness in the household, mother treated violently, divorce or parental separation, and criminal behaviour in the household (Felitti et al., 1998; Burke et al., 2011). With each category counting as one point, with ten categories, the highest possible ACE score is 10 (Felitti et al., 1998; Brown et al., 2009; Felitti, 2010; Narayan et al., 2017; Afifi et al., 2020; Aronson et al., 2020; Finkelhor, 2020; Struck et al., 2021). An additional component of the ACEs was to test the study participant groups for comparability with the original CDC-Kaiser Permanente Study (Felitti et al., 1998). A further comparison was integrated using ACEs replication data carried out within the United Kingdom by Bellis et al. (2015).
• The BCEs scale is a new instrument designed to assess positive early life experiences in adults with a history of childhood adverse experiences. Ostensibly, BCEs are a counterpart to the ACE questionnaire. The BCEs (Narayan et al., 2018) are multiculturally sensitive and applicable, regardless of socioeconomic position, urban-rural background, or immigration status. The BCEs items utilise a developmental psychology framework, integrated with ecological systems theory (Narayan et al., 2017; Crandall, 2019, Crandall, 2020; Merrick et al., 2019; Karatzias et al., 2020; Malti, 2020; Merrick and Narayan, 2020; Starbird and Story, 2020; Doom et al., 2021).
• Each treatment session was timed (minutes) using the metric period recommended by EMDRIA sessions; 60–90 minutes, measuring from commencement of Phase 3 – assessment, to the completion of Phase 7 – closure (including debrief).
• The cost per session (£s/€s) was calculated at £56.49 (€66.36) using economic modelling from the University of Worcester.

After 1 month of each VB2Tr session, another research team member carried out Phase 8 – re-evaluation, conducted a qualitative interview, and obtained 1-month FU data – additional psychometric data were also collected at 6 months.

### Treatment

The research utilised a 1-treatment session intervention (EMDR VB2Tr as a VCP), which was a partial replication of a previous study (Farrell et al., 2020). This study used a beta-tested software programme called remotEMDR^1^, a technology that enabled the delivery of EMDR as a VCP. The remotEMDR is a synchronous programme that offers various visual and acoustic forms of bifocal physical stimulation and includes an integrative video platform, giving EMDR therapists complete control within the session.

The EMDR therapy VB2Tr protocol, adapted for VCP usage, originated from the original B2T (Farrell and Reid, 2020), including pathogenic MV, intensity, and emotionality metrics.

An EMDR Europe Accredited Senior Trainer and Consultant carried out each of the VB2Tr treatment sessions, and each was digitally recorded and made available for treatment fidelity checking. EMDR Europe Consultants and Co-researchers carried out these fidelity checks for the project. The EMDR Foundation Fidelity Rating Scale (EFRS) – version 2 was developed initially by van der Kolk (2007), subsequently revised and updated by Maxfield et al. (2018).

VB2Tr sessions incorporated multiple consent points, including initial recruitment, the commencement of the VB2Tr session, permission to record digitally, and permission to utilise the research participants’ data at the end of the VB2Tr session.

### Statistical Analysis

Statistical analysis utilised the Statistical Package for Social Sciences (SPSS version 26.0; Chicago, IL, United States) to include means and standard deviations calculated for SUD, VOC, MV, ME, and MI before treatment, post-treatment, and at 1-month and 6-month FUs (Table 3). Skewness and kurtosis were estimated in the data sets to evaluate the normality of the outcome measures, and frequencies of total and individual scores for ACEs and BCEs. This detailed examination and alpha testing included generalised estimating equations (GEE) to compare before/after the intervention and FU changes in SUD, VOC, MV, ME, and MI. ACEs and BCEs were added as the covariates in the modelling exercise. The GEE model accounts for time variations and correlations among repeated measurements and does not require the dependent variable to be normally distributed (Locascio and Atri, 2011). Gamma with log link was selected as the outcome variables were skewed. The presence of negative values for SUD, MV, ME, and MI measures was handled by adding a constant value to the data before the analysis. As for descriptive statistics, we used mean ± standard deviation (±SD) for numerical variables and percentage (%) for categoric variables. The *p*-values of <0.05 were considered significant. The overall effect size was calculated using Hedges’ g.

**TABLE 3:** Descriptives – subjective unit of disturbance (SUD) and validity of cognition (VOC): pre, post, 1-month, and 6-month FUs (*N* = 24).

	Pre SUD	Post SUD	SUD-1-mth FU	SUD-6mth FU	Retro VOC Pre	VOC Post	VOC 1-mth FU	VOC 6-mth FU
Mean	7.75	0.17	0.55	0.35	2	6.96	6.86	6.89
Median	8	0	0	0	2	7	7	7
STD	1.39	0.48	0.74	0.59	0.78	0.2	0.35	0.32

## Results

Of the *N* = 24 research participants who took part in the study, all completed the VB2Tr treatment session and post-treatment, 1-month, and 6-month FU measures. There were no dropouts from the study. Tables 3, 4 highlight the descriptive data regarding various measures, namely, SUD, VOC, MV, ME, and MI, and pre, post, 1-month, and 6-month FUs.

**TABLE 4:** Descriptives – memory vividness (MV), memory emotionality (ME), and memory intensity (MI): pre, post, 1-month, and 6-month FUs (*N* = 24).

	Memory vividness			Memory emotionality					Memory intensity			
	Pre	Post	1-mth FU	6-mth FU	Pre	Post	1-mth FU	6-mth FU	Pre	Post	1-mth FU	6-mth FU
Mean	8.04	1.42	0.227	0.579	8.33	0.417	0.0909	0.211	8.46	0.0417	0.409	0.474
Median	8	0	0	0	8	0	0	0	8	0	0	0
Standard deviation	1.78	2.41	1.82	1.46	1.43	0.717	2.2	1.23	1.41	0.999	1.99	1.68
Minimum	5	0	−7	−3	6	0	−8	−4	6	−4	−6	−6
Maximum	10	8	2	2	10	2	3	1	10	2	1	1

**FIGURE 1:**
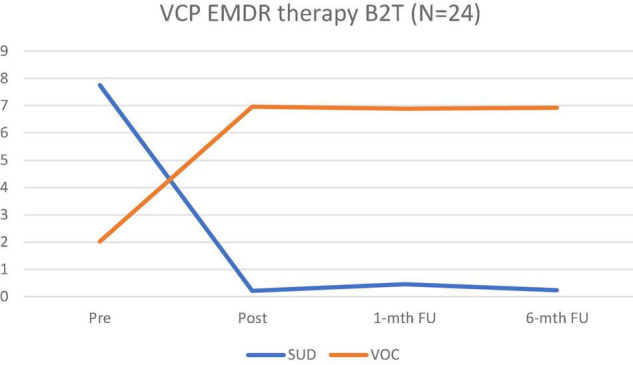
Alterations in SUD and VOC scores at pre, post, 1-month, and 6-month FUs.

Hypothesis 1. Figure 1 shows the decrease in SUD and the increase in the VOC at the post, 1-month, and 6-month FUs.

Additionally, Table 5 highlights the mean, standard deviation, skewness, kurtosis, baseline, and *p* values for the SUD and VOC at pre, post, 1-month, and 6-month FUs and the maintenance of the VB2Tr treatment effect in more detail.

**TABLE 5:** Means, SD, skewness, and kurtosis for SUD and VOC at pre, post, 1-month, and 6-month FUs.

	Mean (SD)	Skewness (SE)	Kurtosis (SE)	B (SE)	*p* value
Pre-SUD	7.75 (1.39)	−0.15 (0.47)	−0.95 (0.92)	0	
Post-SUD	0.21 (0.49)	2.72 (0.47)	7.73 (0.92)	−1.02 (0.03)	<0.001*
1 m FU SUD	0.64 (0.79)	0.78 (0.49)	−0.89 (0.95)	−0.94 (0.05)	<0.001*
6mFU SUD	0.23 (0.95)	−1.74 (0.51)	6.99 (0.99)	−0.99 (0.01)	<0.001*
Pre-VOC	2.02 (0.79)	−0.08 (0.47)	−1.36 (0.92)	0	
Post-VOC	6.96 (0.20)	−4.90 (0.47)	24.00 (0.92)	1.22 (0.07)	<0.001*
1 m FU VOC	6.87 (0.31)	−2.60 (0.49)	5.63 (0.95)	1.21 (0.08)	<0.001*
6 m FU VOC	6.92 (0.25)	−3.34 (0.52)	11.19 (1.01)	1.22 (0.08)	<0.001*

*Statistically significant.

There was a substantial reduction in SUD after receiving EMDR in comparison with the baseline assessment (*B* = −1.02, *SE* = 0.03, *p* < 0.001). The decrease in SUD was maintained in the FU assessments at 1 month (*B* = -0.94, *SE* = 0.05, *p* < 0.001) and 6 months (*B* = -0.99, *SE* = 0.04, *p* < 0.001) in comparison with the baseline. Simultaneously, there was an increase in VOC after receiving EMDR in comparison with the baseline assessment (*B* = 1.22, *SE* = 0.07, *p* < 0.001). The increase was maintained after 1-month (*B* = 1.21, *SE* = 0.08, *p* < 0.001) and 6-month (*B* = 1.22, *SE* = 0.08, *p* < 0.001) post intervention.

The results of this study indicate that VB2Tr decreased the SUD and increased the VOC in the treatment of a pathogenic memory tested at pre, post, 1-month, and 6-month FUs, suggesting that using VB2Tr as a VCP demonstrated a treatment effect on the pathogenic memory when measured by the SUD and VOC. Furthermore, there was a statistically significant difference between pre-treatment (*M* = 7.75, SD = 1.39) and 6-month FU (*M* = 0.35, SD = 0.59), with a Hedges’ *g* effect size value (*g* = 6.71) suggesting high practical significance. Therefore, we rejected the directional hypothesis that there is no difference in the SUD or VOC when using the VB2Tr EMDR intervention as a VCP.

**FIGURE 2:**
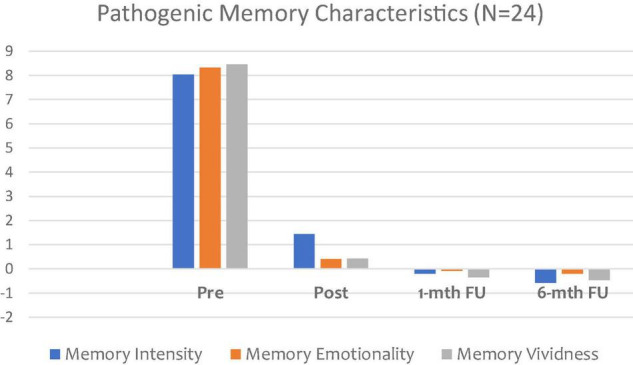
Changes in pathogenic memory subjective characteristics.

Hypothesis 2. Figure 2 shows a reduction in the nature and characteristics of the pathogenic memory, including MV, emotionality, and intensity. For some research participants, alterations in memory characteristics indicated positive change rather than disturbance (negative), and therefore positive change is presented as a minus score.

As Figure 2 demonstrates, the VB2Tr intervention clearly impacted on the three areas of pathogenic distinctiveness, namely, MV, emotionality, and intensity, with results maintained at both 1-month and 6-month FU. There were significant decreases in MV (*B* = -0.42, SE = 0.05, *p* < 0.001), ME (*B* = -0.61, SE = 0.03, *p* < 0.001), and MI (*B* = -0.77, SE = 0.04, *p* < 0.001) following the intervention. These effects were maintained in the first month FU for ME (*B* = -0.65, SE = 0.06, *p* < 0.001), MI (*B* = -0.79, SE = 0.03, *p* < 0.001), MV (*B* = -0.60, SE = 0.07, *p* < 0.001) and sustained after 6 months for MV (*B* = -0.58, SE = 0.03, *p* < 0.001), ME (*B* = -0.62, SE = 0.03, *p* < 0.001), MI (*B* = -0.79, SE = 0.04, *p* < 0.001). Overall, these results indicate changes that were consistently, statistically significant at *p* < 0.001. Additionally, the results demonstrate a favourable dose effect, with potential evidence in support of resilience and post-traumatic growth, as indicated by the treatment effect emphasis between pre and 6-month FU. This represents a significant finding from this study.

Therefore, it would be reasonable to assume that when VB2Tr is delivered as a VCP, it has the potential to instigate distinct changes to core components of the pathogenic memory, suggesting evidence of memory reconsolidation. Regarding hypothesis 2, distinctiveness – VB2Tr, as a VCP, will have no impact in reducing MI, ME, and MV of a pathogenic memory following intervention when measured at post-treatment, 1-month, and 6-month in comparison with a pre-measure. Therefore, the directional hypothesis is not supported.

**FIGURE 3:**
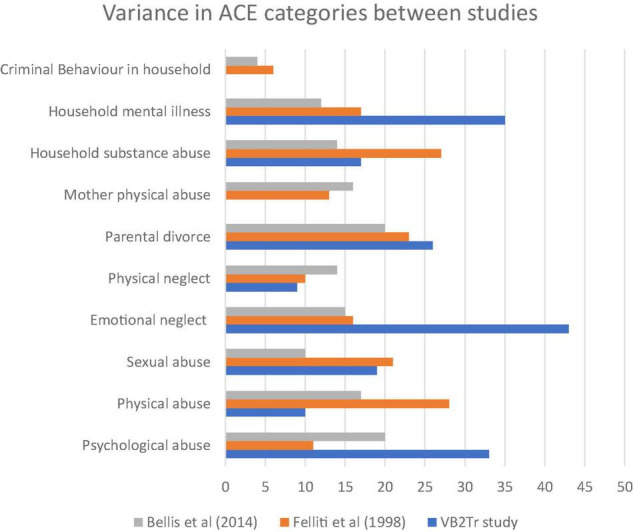
ACE by category between studies.

**FIGURE 4:**
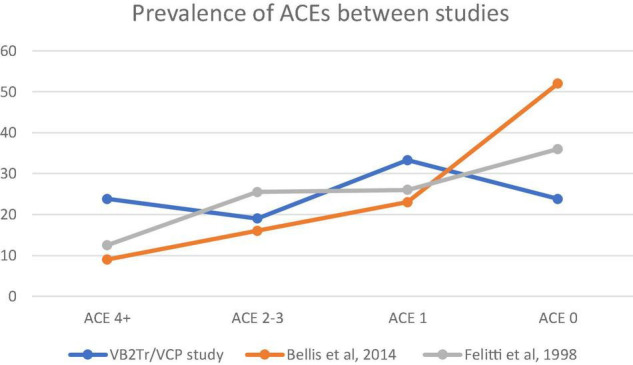
Prevalence of ACE scores between the three groups: VB2Tr.

Hypothesis 3. Figures 3, 4 demonstrate that exposure to ACEs or BCEs did not influence the processing of the pathogenic memory or the intervention outcome following the utilisation of VB2Tr.

As explained previously, testing the relevance hypothesis compared the study participant group with the original primary studies (Felitti et al., 1998; Bellis et al., 2014a,b, 2015, 2017, 2019). A single factor ANOVA explored the between-group variances. This one-way analysis of variance is a technique used to compare two or more samples when utilising numerical or categorical data.

Figure 4 highlights the prevalence of ACEs between studies (Felitti et al., 1998; Bellis et al., 2014b). A descriptive review of the results suggests higher exposure to 4 + ACEs within the VB2Tr participant group; however, results yielded an *F*(3,4) = 8.45, *p*-value < 0.03*, suggesting that there were, in fact, differences between the three groups in terms of the prevalence of ACEs, therefore, the directional hypothesis is not supported.

Table 6 provides more descriptive data about the VB2Tr participant group relating to specific exposure to ACEs. The results focussed on exposure to physical abuse, physical neglect, mother physical abuse, and criminal behaviour in the household.

**TABLE 6:** Types of adverse childhood experiences (ACE).

ACEs	Incidence	Sig. (2-test)
Psychological abuse	7 (33%)	0.189
Physical abuse	2 (10%)	<0.001*
Sexual abuse	4 (19%)	0.007
Emotional neglect	9 (43%)	0.664
Physical neglect	2 (9%)	<0.001*
Parental divorce	6 (26%)	0.035
Mother physical abuse	0 (0%)	<0.001*
Household substance abuse	4 (17%)	0.003
Household mental illness	8 (35%)	0.210
Criminal behaviour in household	0 (0%)	<0.001*

*p < 0.001.

**FIGURE 5:**
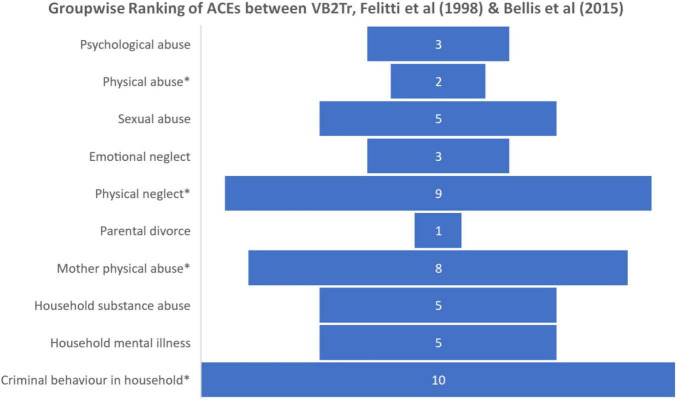
Groupwise ranking of ACEs between groups, *p < 0.001.

Although an ANOVA revealed a distinction between the three groups, it is essential to highlight the elevated incidence of exposure to psychological abuse, emotional neglect, household mental illness, absence of exposure to mother physical abuse, and criminal behaviour in the household VB2Tr research participant group. Figure 5 ranks the scores of the ten questions of the original ACEs questionnaire from most prevalent (1) to least (10).

**FIGURE 6:**
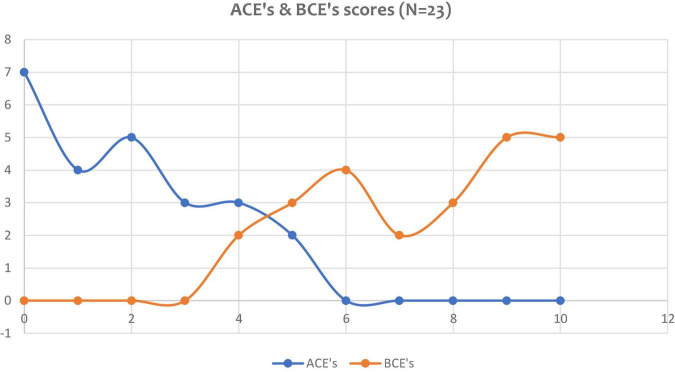
Frequency of ACE and BCE scores of the research participants.

Figure 6 compares the frequency of both ACEs and BCEs from the VB2Tr research population. The mean ACE was 1.8 (SD 1.68) and the BCE was 7.6 (SD 2.06). The correlation between ACEs and BCEs is *r*(22) = -0.48.

For the BCEs, the mean for the sample was 7.6 (*SD* = 2.06), and the median was 8. As shown in Table 7, most participants reported having eight (17.4%), nine (21.7%), and ten (21.7%) benevolent experiences in their childhood. All participants reported at least four BCEs. Table 5 displays the frequency of each type of BCEs in the current sample. Results suggest that having “At least one good friend” (96%) and “Opportunities to have a good time” (91%) were the most frequent benevolent experiences.

**TABLE 7:** Types of BCEs.

BCEs	Incidence	Sig. (2-test)
At least one caregiver with whom you felt safe	18 (78%)	0.011
At least one good friend	22 (96%)	<0.001*
Beliefs that gave you comfort	16 (70%)	0.093
Enjoyment at school	18 (78%)	0.011
At least one teacher that cared	20 (87%)	<0.001*
Good neighbours	17 (74%)	0.035
An adult (not a parent/caregiver or the person from *11) who could provide you with support or advice	12 (52%)	1.000
Opportunities to have a good time	21 (91%)	<0.001*
Like yourself or feel comfortable with yourself	12 (52%)	1.000
Predictable home routine, like regular meals and a regular bedtime	20 (87%)	<0.001*

*Statistically significant.

In exploring further into hypothesis 3, relevance – first, results seem to indicate that neither ACEs nor BCEs scores did not influence the outcome of the intervention. Second, although 75% of the research participants admitted their motivation for participation was based firmly on “non-disclosure,” results indicated 87.5% did disclose their target memory to the treating clinician. Of these 87.5%, Table 8 highlights the categories of target memories disclosed.

**TABLE 8:** Disclosed target memory themes and frequencies chosen by research participants for VB2Tr as a VCP.

° Sexual assault (3)
° Child abuse (4)
° Parental neglect (1)
° Fatal road traffic collision (1)
° Occupational bullying (4)
° Complicated grief (2)
° Episodes involving shame and humiliation (6)

As indicated earlier, using VB2Tr demonstrates a distinct treatment effect with Table 8, highlighting clinical applicability. In addition, desensitisation and reprocessing of these trauma memories occurred irrespective of either ACEs or BCEs. These results were consistent at 1-month and 6-month FU. Consequently, this data set supports the assertion of hypothesis 3, therefore, the directional hypothesis is not supported in relation to ACEs and BCEs.

In testing hypothesis 4, efficiency – the administration of VB2Tr was tested against the period recommended by EMDRIA sessions; 60–90 min. Results for this study used a time metric (minutes) from the commencement of Phase 3, assessment, to the completion of Phase 7, closure (including debrief). Of the *N* = 24 research participants, the average VB2Tr session was 57 min and 27 s, with an SD of 17 min 27 s. Results highlight that the treatment sessions were below the 60 min threshold.

**FIGURE 7:**
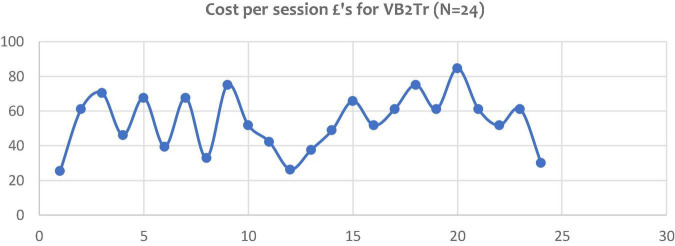
Variance in cost of VB2Tr treatment sessions carried out as a VCP.

Testing the costing element of hypothesis 4 required economic modelling using University of Worcester financial algorithms. The UW costing model used for each VB2Tr treatment session was calculated at £56.49 (€66.36). Of the 24 clinical sessions of VB2Tr carried out, the mean cost per session was £54.02 (€63.45). This represented potential modest savings of £2.47 (€2.91) per session. However, Figure 7 indicates the variation in treatment costs for each individual session. One of the distinct advantages of remote intervention is the reduction in client-related costs such as travel time, transportation, and parking.

Results indicate savings in terms of time and efficiency, with additional health economic benefits. The results of hypothesis 4 results suggest the rejection of the directional hypothesis.

## Discussion

The rationale for this study was to ascertain how EMDR therapy could be used as a VCP considering the current COVID-19 pandemic, where social distancing is a vital strategy in reducing infection rates. However, more specifically, this research wanted to explore the potential use of the EMDR therapy VB2Tr protocol virtual version as a VCP, in order to determine its fitness for purpose, distinctiveness, relevance, and efficiency.

The memory targets identified in Table 8 suggest that the research participants worked on distressing pathogenic memories of major adverse life events and that the intervention (VB2Tr) suggests a treatment effect. Despite a large effect size (Hedges’ *g* = 6.71), this should be regarded with caution to not overgeneralise. As the primacy of this research was to demonstrate “proof of concept,” the evidence from this study suggests potential considerations for both scalability and progression to a clinical population with a distinct diagnosis such as PTSD or complex PTSD. For this reason, future research should utilise both an experimental design and a clinical population, both of which would potentially deliver a more realistic treatment effect size.

To the best of our knowledge, this is the first EMDR therapy study that has examined both ACEs and BCEs. The most significant ACEs were emotional neglect, exposure to household mental illness, and psychological abuse up to 18 years of age. However, exposure to physical abuse by the mother and criminal behaviour in the household, with exposure to physical abuse and physical neglect, presented as the more prevalent ACEs in this research population.

The BCEs highlighted positive early life experiences in adults who built resilience and provided a counterbalance to ACEs. These positive childhood experiences include effective caregiving, quality parenting, close relationships with other significant adults, effective schooling, and community. Higher BCEs are associated with more favourable long-term development (Masten, 2014). Within the participant group, the most decisive factors included having at least one good friend, at least one teacher who cared, opportunities to have a good time, and a predictable home routine. Although the data set indicates high levels of BCEs, caution is required as the study participants were highly trained mental health professionals and are not a clinical population. Other interesting observable aspects from the dataset highlight a dichotomy between having an adult (other than a caregiver) who could support and advice and liking or feeling comfortable with oneself. More research is needed to understand further the impact that ACEs and BCEs have on and explore further if and how BCEs act as a potential resilience to counteract the impact of ACEs. However, the results of this study highlight that trauma processing occurred using VB2Tr irrespective of the research participants’ ACE or BCE scores.

Regarding testing hypotheses 1 through 4, the results from the study indicate a treatment effect from using the remotEMDR software to carry out VB2Tr as a VCP. Although the alterations in SUD and VOC are highly consistent with the more comprehensive empirical support for EMDR therapy, the data highlighting personal changes to the characteristics of the pathogenic memory targeted from processing is undoubtedly intriguing. Again, caution is necessary as the sample size is relatively small and would need further testing with a clear clinical population with a more formal medico-legal diagnosis.

In testing hypothesis 3, relevance, the results for disclosure (90%) appear consistent with the previous B2T study in Northern Iraq (Farrell et al., 2020), highlighting a clear clinical benefit in using both B2T and VB2Tr EMDR therapy protocols. This VB2Tr study adds to the literature demonstrating equal effectiveness and suggests a potential correlation between non-disclosure and the level of SUD. Desensitisation and reprocessing of the pathogenic memory increase the probability of disclosure with results of 87.5% for this VB2Tr study. The clinical advantages of this make this an effective tool for use as a trauma treatment intervention, highlighting a distinct benefit of using EMDR therapy for undisclosed trauma memories compared to other trauma-focussed interventions. Again, caution is required as further research is needed to investigate this aspect.

The use of three additional subjective measures, namely, MI, MV, and ME, within this study suggests an argument for including these within the EMDR therapy B2T and the VB2Tr protocol, but also within the standard protocol. These measures appear particularly useful in understanding the subjective experience of the trauma memory targeted for processing. Further research and investigation are needed to pursue this argument further.

Although this was only a 1-treatment session study, the data set reveals an interesting health economic argument, with an average session cost of £56.49 (€66.36). Although the study yielded modest economic savings of £2.47 (€2.91) per session, the cumulative implications of this, in addition to the clinical benefits, suggest a particularly compelling argument.

As indicated earlier, there are two major flaws in this study. First, the research participant group, although frontline mental health workers working under extraordinary circumstances, is not a non-clinical population. However, in mitigation, this is a “proof of concept” study. Second, relating to methodology, this was a straightforward pre-test–post-test research design. A future study would need to utilise a more robust experimental framework. However, results from this study suggest a more empirical foundation upon which future studies can build. As the study involved non-disclosure of trauma memory, randomisation was not possible for both ethical and moral reasons. Scaling up to a full randomised control study would not be possible; however, a quasi-experimental design would be a viable alternative.

To summarise, the research results suggest VB2Tr EMDR therapy to be an effective, fit-for-purpose, safe-to-use trauma treatment intervention. Additionally, the results highlight its clinical relevance and applicability as a trauma intervention. Furthermore, the remotEMDR software provided a highly effective platform for delivering EMDR therapy as a VCP. Although the researchers acknowledge that other platforms exist, results from this study are based entirely on remotEMDR.

## Conclusion

This research study demonstrated encouraging evidence in support of EMDR therapy as VCP in treating a pathogenic (trauma) memory. In addition, the study explored specific factors influenced by the treatment intervention. The results of this study highlight the potential of using EMDR therapy, in this case using the B2T protocol as a VCP. Furthermore, results suggest the intervention has clinical applicability. Caution does need to be exercised regarding both the lack of a clinical population and the need for a more experimental design. However, results from this study demonstrate “proof of concept.”

However, to have an intervention that appears effective with either shame-based or fear-based trauma memories suggests great potential regarding clinical applicability. To have such an intervention that works on trauma memories that clients are unwilling to disclose due to fear, blame, or prejudice suggests distinct advantages for EMDR therapy in the repertoire of empirically supported trauma treatment interventions. Furthermore, to have such an intervention that appears safe and effective adds more temerity to this assertion. This study highlights how the VB2Tr EMDR therapy scripted protocol alters core characteristics of the pathogenic memory itself, including memory disturbance, emotionality, intensity, and vividness. The results also demonstrate that these changes occur irrespective of either ACEs or BCEs. Another critical finding relates to resilience and post-traumatic growth factors more powerful when considering a stark choice for clients, such as disclosure of the memory or no treatment, they choose no treatment. Providing a credible alternative in this critical decision-making juncture suggests distinct clinical benefits and applicability. Potential health efficiency arguments highlighted by this research are tentative yet worthy of further investigation and critical consideration.

It does need to be acknowledged that psychological treatment through the medium of videoconferencing may not suit some people. These reasons may be technological, safety factors, security, and/or individual choice. Understandably, this must be respected and accommodated. This study highlights that when individuals are given a choice between “disclosure” or “no treatment,” the research participants in this study chose the latter and not the former. This raises an interesting ethical question: is it better to do something, than nothing? Being able to offer clients greater choice is essential. In a world of increasing uncertainty and insecurity, the need for having suitable, evidence-based alternatives to “in-person” is paramount.

In summary, these results demonstrate proof of concept and put forward the case for further research and investigation. The stage 2 aspect of the study will further test the EMDR intervention as a VCP with a defined, clinical control group. The research supports the case for EMDR therapy as a credible treatment when used as a VCP.

The COVID-19 pandemic has challenged existing mental health and psychology services enormously. As the global burden of psychological trauma continues unabated, and we remain in an environment of scarcity in resources, any intervention that provides distinct choice and effectiveness in treating shame- or fear-based memories is a compelling argument, and much-needed treatment approach. The EMDR therapy “Blind 2 Therapist” appears to be a distinctly helpful psychotherapeutic tool in this endeavour. The fact that such an intervention has the potential health efficiency benefits strengthens this argument further. Any intervention, virtual or not, which improves accessibility and reach must be welcomed.

## Data Availability Statement

The EMDR therapy Virtual Blind 2 therapist (VB2Tr) protocol used for the study is available at: https://trnireland.ie/. Additional original contributions presented in the study are included in the article/supplementary material, further inquiries can be directed to the corresponding author.

## Ethics Statement

The studies involving human participants were reviewed and approved by the University of Worcester, Worcester, United Kingdom. The patients/participants provided their written informed consent to participate in this study.

## Author Contributions

DF conceived the study, carried out the VB2Tr treatment sessions, and was the chief investigator for the project. LK, PM, and ZZ were part of the research team, acquired the FU date, and conducted the qualitative interviews. AF, DF, and MK carried out the data analysis. The primary author was the principal investigator. All authors contributed to the overall article and approved the submitted version for publication.

## Conflict of Interest

NG is the co-founder and CEO of remotEMDR. The remaining authors declare that the research was conducted in the absence of any commercial or financial relationships that could be construed as a potential conflict of interest.

## Publisher’s Note

All claims expressed in this article are solely those of the authors and do not necessarily represent those of their affiliated organizations, or those of the publisher, the editors and the reviewers. Any product that may be evaluated in this article, or claim that may be made by its manufacturer, is not guaranteed or endorsed by the publisher.

## Footnotes

1
www.remotEMDR.com
